# Transcatheter Caval Valve Implantation for Tricuspid Regurgitation After Single Leaflet Device Attachment

**DOI:** 10.1016/j.jaccas.2022.02.014

**Published:** 2022-04-20

**Authors:** Andi Rroku, Fabian Barbieri, Ulf Landmesser, Carsten Skurk, Mario Kasner, Markus Reinthaler

**Affiliations:** a Charité–Universitätsmedizin Berlin, corporate member of Freie Universität Berlin and Humboldt-Universität zu Berlin, Department of Cardiology, Berlin, Germany; b DZHK (German Centre for Cardiovascular Research), partner site Berlin, Berlin, Germany; c Berlin Institute of Health at Charité–Universitätsmedizin Berlin, Berlin, Germany; d Institute of Active Polymers and Berlin-Brandenburg Center for Regenerative Therapies, Helmholtz-Zentrum Hereon, Teltow, Germany

**Keywords:** bicaval valve implantation, case report, single leaflet device attachment, transcatheter edge-to-edge repair, tricuspid regurgitation, CAVI, caval valve implantation, IVC, inferior vena cava, LVEF, left ventricular ejection fraction, SLDA, single leaflet device attachment, SVC, superior vena cava, TEER, transcatheter edge-to-edge repair, TR, tricuspid regurgitation

## Abstract

An 86-year-old patient experienced progressive heart failure symptoms. Echocardiographic evaluation revealed severe tricuspid regurgitation, which was treated by transcatheter edge-to-edge repair. During the procedure, single leaflet device attachment occurred. On the basis of a prohibitive surgical risk, caval valve implantation was performed, with no notable complications. (**Level of Difficulty: Advanced.**)

## Central Illustration

**Figure:**
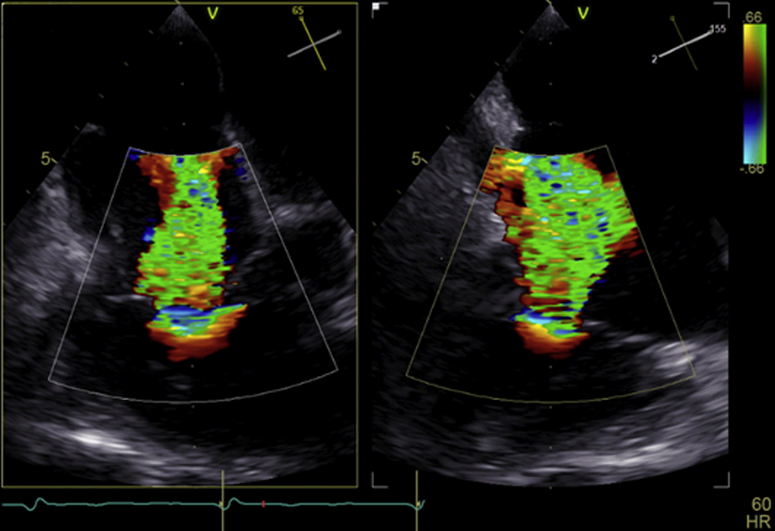

## History of Presentation

An 86-year-old man presented to our cardiology department with worsening dyspnea (New York Heart Association functional class III), fatigue, peripheral edema, and signs of central congestion.

**Learning Objectives**

• To identify single leaflet device attachment with consecutive worsening of tricuspid regurgitation as a severe complication with limited therapeutic options other than surgical valve repair/replacement.
• To consider caval valve implantation as a useful alternative for the treatment of severe symptomatic tricuspid regurgitation in patients with prohibitive surgical risk and previously failed transcatheter edge-to-edge repair.

## Past Medical History

The patient was well known at our department due to his history of ischemic cardiomyopathy with surgical aorto-coronary venous bypass grafting being performed in 1995 and subsequent repetitive percutaneous interventions. In the setting of heart failure with reduced ejection fraction (left ventricular ejection fraction [LVEF]: 25%) and optimized medical therapy, an implantable cardioverter-defibrillator was implanted with the purpose of primary prophylaxis. Later on that year, after the patient experienced recurrent hospitalizations due to heart failure, severe secondary mitral regurgitation was treated by transcatheter edge-to-edge repair (TEER) with implantation of 2 clips. Further known comorbidities included arterial hypertension, dyslipidemia, chronic obstructive pulmonary disease, and chronic renal failure. Current presentation at our emergency department was ∼1 year after the last cardiovascular intervention.

## Differential Diagnosis

Given the patient’s medical history, differential diagnosis included congestive heart failure caused by worsening of LVEF and/or right ventricular function, deterioration of any of his heart valves, pacing-induced cardiomyopathy, atrial fibrillation, or progressive kidney failure.

## Investigations

**Figure 1:**
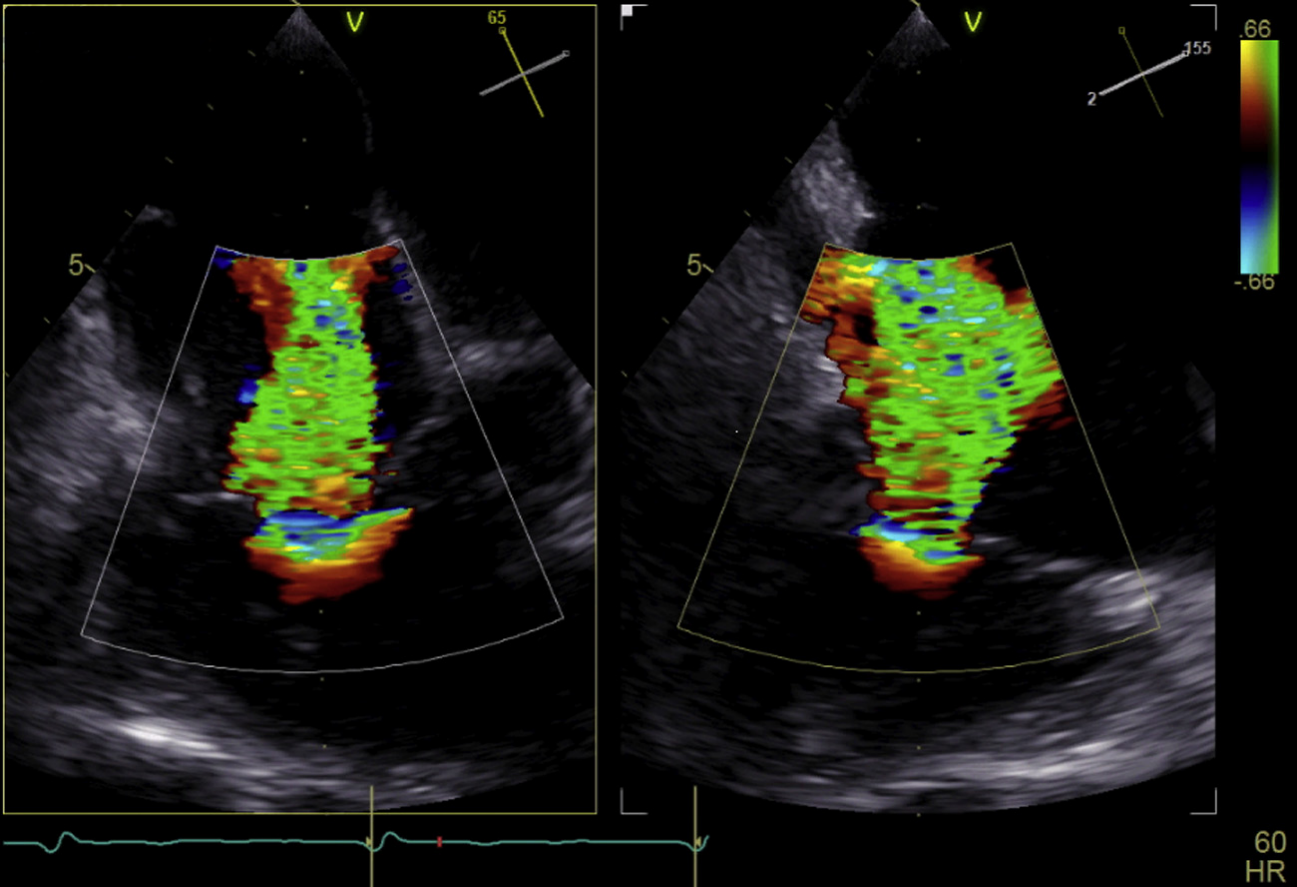
Biplane Echocardiography Showing Massive Tricuspid Regurgitation

Transthoracic echocardiography confirmed known impairment of LVEF as well as severe bi-atrial dilatation. Right ventricular function was marginally reduced, with a tricuspid annular plane excursion of 16 mm, and calculated systolic pulmonary arterial pressure was ∼55 mm Hg. In addition to moderate residual mitral regurgitation, transesophageal echocardiography confirmed massive functional tricuspid regurgitation (TR) (vena contracta: 15 × 13 mm) (Figure 1) with corresponding annular dilation (antero-posterior: 48 mm; mediolateral: 53 mm). The jet was located mainly in the central area of the valve but also extended into the anteroseptal commissure. The calculated EuroSCORE II estimated a 30-day mortality risk of 10.6%.

## Management

After an intensified intravenous diuretic treatment regimen, the patient remained symptomatic, and massive TR was still present. Further treatment options were discussed in the interdisciplinary heart team. Due to the high-surgical-risk constellation, surgical repair/replacement was abandoned, and interventional therapies were discussed. Decision was made to opt for TEER of the tricuspid valve by off-label usage of MitraClip XTR devices (Abbott Laboratories) because screening for interventional annuloplasty failed owing to proximity of the right coronary artery.

**Figure 2:**
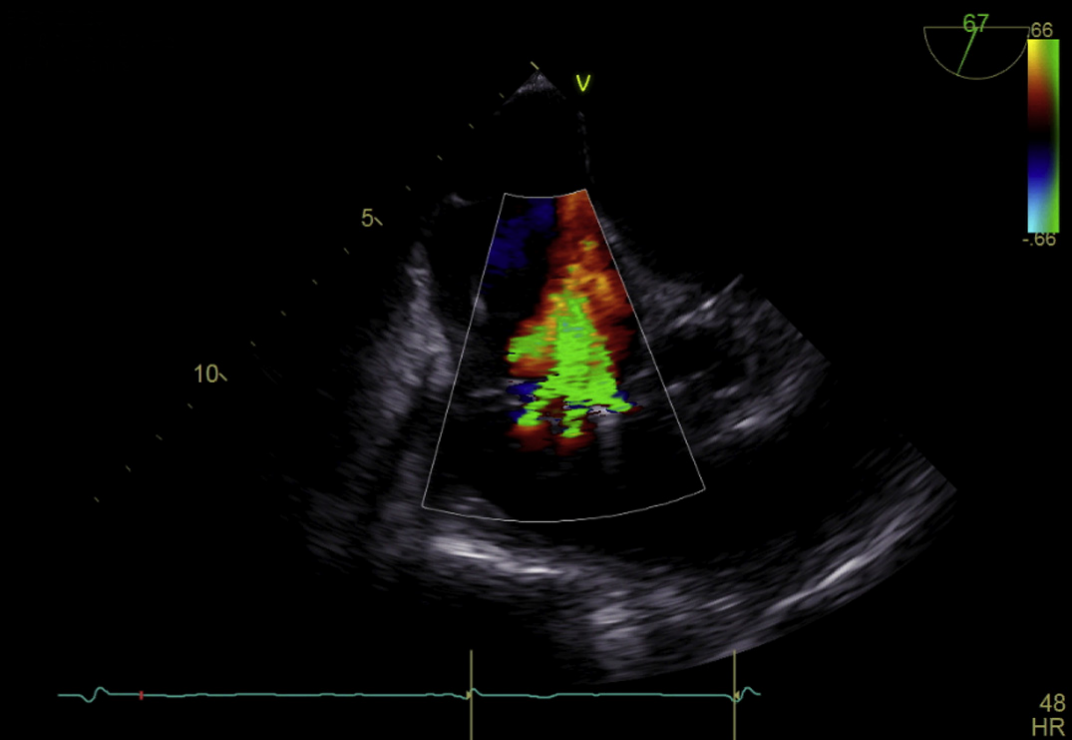
Echocardiography Showing Severe Tricuspid Regurgitation After Single Leaflet Device Attachment

Ten days after admission, the procedure was conducted under general anesthesia. Implantation of the first device in the anteroseptal commissure led to reduction of TR (Videos 1 and 2). Further reduction was intended by implantation of a second device in the posteroseptal location (Video 3). After release of the device (Video 4), a detachment of the septal leaflet occurred, causing single leaflet device attachment (SLDA) (Videos 5 and 6). However, despite the detached clip, mild improvement to severe residual TR (Figure 2) was observed. The procedure was terminated at this point owing to missing target options. The patient was transferred to the ward after observation at our intensive care unit without any notable complications and discharged on the sixth postoperative day. Before discharge, transthoracic echocardiography confirmed severe TR, while complete detachment of the device was excluded.

Two weeks later, during an unscheduled visit because of orthostatic syncope, the patient described an ameliorated symptomatic status with regressive peripheral edema.

Unfortunately, symptoms deteriorated again over upcoming months. Worsening to torrential TR was confirmed as suspected, despite intensified oral diuretic therapy. Remaining treatment options were discussed with the patient. Given his momentary improvement in quality of life after the first procedure and the presence of chronic injury of liver parenchyma (interpreted as cirrhosis cardiaque), the patient agreed to evaluation for caval valve implantation (CAVI). After confirmation of eligibility within a compassionate-use program, an interventional heterotopic tricuspid valve replacement was eventually planned. Preoperative planning included computed tomography angiography, right-heart catheterization (systolic pulmonary arterial pressure: 47 mm Hg; V-wave: 27 mm Hg; and mean right atrial pressure: 18 mm Hg), as well as an angiographic assessment of the inferior vena cava (IVC) and superior vena cava (SVC) to define the designated landing zones.

The intervention was again conducted under general anesthesia. Transfemoral endovascular access was achieved, and a pigtail catheter was positioned within the right pulmonary artery.

**Figure 3:**
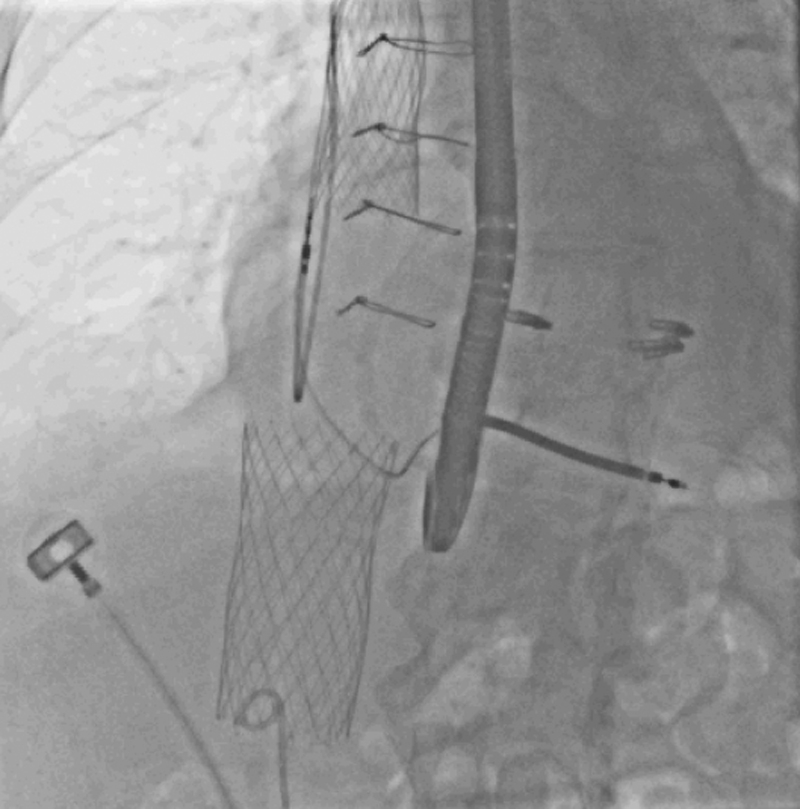
Fluoroscopy After Caval Valve Implantation

Following introduction of the sheath (27.5-F), the delivery catheter was inserted over a stiff wire, while the tip was positioned beneath the brachiocephalic and azygos vein confluence. Controlled top-down deployment of the SVC prosthesis was executed, and valve functionality was tested via angiography (Video 7). Similarly, the IVC valve was carefully positioned and deployed (Video 8). Final echocardiographic examination and angiography yielded a good result for both valves without any notable complications (Figure 3, Videos 9 and 10).

## Discussion

Severe symptomatic TR constitutes a growing burden with major impact on morbidity and mortality.^1^ Despite the high prevalence of TR, isolated surgical repair is often withheld from patients because of unsatisfactory perioperative mortality.^2^ Current approaches for interventional treatment of TR provide a good safety profile,^3^ but long-term data on patient outcomes remain sparse.^4^ Although first data look promising, periprocedural complications occur and may necessitate re-intervention. One of these complications is SLDA. SLDA is reported in up to 7.7% of patients undergoing TEER for TR and is mainly caused by insufficient leaflet grasping, which was retrospectively the reason in our case.^3^ Nonetheless, it may also occur after adequate grasping and is then typically caused by leaflet tear or perforation.^5^ Due to the fact that TEER is predominantly performed in high-risk or inoperable patients, alternative treatment options are limited whenever SLDA occurs. Currently, there is only 1 case report published describing stabilization of SLDA by implantation of another device.^6^

To our knowledge, we present the first case report of tricuspid valve SLDA resolved by CAVI. Transcatheter CAVI is a rather new treatment option for patients with symptomatic TR, who are unfavorable candidates for open surgery and other structural interventions. The concept focuses on preventing regurgitation flow within the SVC and IVC and therefore aims to reduce hepatic, abdominal, and systemic venous congestion and their associated symptoms such as ascites and peripheral edema. In addition, previous publications also noted an occurrence of right ventricular remodeling caused by reduction in volume load, which ultimately increased stroke volume and cardiac output. At the chronic phase, reduction in hepatic congestion also ought to decrease the risk of cardiac cirrhosis.^7^^,^^8^ Due to its noninterfering nature with the valvular apparatus, CAVI presents a valuable bail-out option after SLDA. This was especially relevant in our case as there was no suitable target area available for placement of an additional device.

Several devices are or have been under clinical investigation:

1. The Sapien XT/3 (Edwards Lifesciences) has been used for intracaval implantation, being positioned after preceding stent implantation. A randomized controlled trial evaluating its effect against optimal medical therapy was stopped due to an unexpectedly high rate of valve dislocations.^9^
2. The Tricento transcatheter heart valve (NVT) is a custom-made self-expanding stent with landing zones in the SVC and IVC and a bicuspid valve opening in the lower atrial segment of the stent.^10^ Its safety and performance will be investigated in the TRICAR trial (Investigation of a Transcatheter Tricuspid Valved Stent Graft in Patients With Carcinoid Heart Disease; NCT05064514).
3. The TricValve (P+F Products + Features GmbH), which was also used in our case, is a dedicated self-expandable CAVI device with 2 nonidentical tissue valves on a nitinol belly-shaped stent positioned in the SVC and IVC.^7^^,^^8^ The TRICUS STUDY EURO (Safety and Efficacy of the TricValve® Transcatheter Bicaval Valves System in the Superior and Inferior Vena Cava in Patients With Severe Tricuspid Regurgitation; NCT04141137), which aims to investigate safety and performance in a single-arm experimental study, is ongoing.

## Follow-up

After a prolonged intensive care treatment due to pneumonic sepsis with respiratory insufficiency, our patient was transferred to a heart failure rehabilitation center. Last echocardiographic assessment yielded an adequate caval valve function with no signs of regurgitation, while right ventricular function remained unchanged (tricuspid annular plane excursion: 16 mm).

## Conclusions

Patients who experience SLDA in the course of TEER are often left untreated because of technical and/or anatomical limitations. CAVI seems to be a feasible bail-out option in patients experiencing recurrent TR with progressing symptoms and at a high surgical risk.

## Funding Support and Author Disclosures

This retrospective report was approved by the Ethics Committee of Charité–Universitätsmedizin Berlin (EA4/013/21). Dr Barbieri has received grant support from Abbott Laboratories and Boston Scientific; and consulting fees from Boston Scientific. Dr Landmesser has received personal fees from Abbott Laboratories, Biotronik, and Boston Scientific. All other authors have reported that they have no relationships relevant to the contents of this paper to disclose.

## Appendix

Supplemental Video 1 Preprocedural Short-Axis View With Color Doppler

Supplemental Video 10 Color Doppler After IVC Valve Implantation

Supplemental Video 2 Short-Axis View After First Clip

Supplemental Video 3 Short-Axis View With Color Doppler After Second Clip

Supplemental Video 4 Biplane View After Second Clip

Supplemental Video 5 Biplane View After SLDA of Second Clip

Supplemental Video 6 Short-Axis View With Color Doppler After SLDA

Supplemental Video 7 Fluoroscopy of CAVI (SVC)

Supplemental Video 8 Fluoroscopy of CAVI (IVC)

Supplemental Video 9 Color Doppler After SVC Valve Implantation
